# *Trans*-*p*-Coumaryl Alcohol as a Bioactive Compound and Anti-Inflammatory Agent in Wannachawee Recipe for Psoriasis

**DOI:** 10.3390/pharmaceutics17070864

**Published:** 2025-06-30

**Authors:** Supreeya Tantipat, Kongkiat Trisuwan, Phraepakaporn Kunnaja, Seewaboon Sireeratawong, Surapol Natakankitkul, Surasak Imiam, Sunee Chansakaow

**Affiliations:** 1 Department of Pharmaceutical Sciences, Faculty of Pharmacy, Chiang Mai University, Chiang Mai 50200, Thailand; yayee.ttp13@gmail.com (S.T.); surapol_nat@cmu.ac.th (S.N.); 2 Department of Chemistry, Faculty of Science, Chiang Mai University, Chiang Mai 50200, Thailand; kongkiat.t@cmu.ac.th; 3 Center of Excellence for Innovation in Chemistry, Chiang Mai University, Chiang Mai 50200, Thailand; 4 Department of Medical Technology, Faculty of Associated Medical Sciences, Chiang Mai University, Chiang Mai 50200, Thailand; phraepakaporn.k@cmu.ac.th; 5 Department of Pharmacology, Faculty of Medicine, Chiang Mai University, Chiang Mai 50200, Thailand; seewaboon@gmail.com; 6 Clinical Research Center for Food and Herbal Product Trials and Development (CR-FAH), Faculty of Medicine, Chiang Mai University, Chiang Mai 50200, Thailand; 7 Department of Thai Traditional Medicine, Phrapokklao Hospital, Chanthaburi 22000, Thailand; surasak.alexx@gmail.com

**Keywords:** Wannachawee recipe, psoriasis, anti-inflammatory activity, *p*-coumaryl aldehyde, *trans*-*p*-coumaryl alcohol

## Abstract

**Background/Objectives**: Wannachawee recipe (WCR) has been listed in the Hospital Traditional Medicine Formulary and has been used as a Thai medicine to treat psoriasis in the Thai Traditional Medicine Clinic of Prapokklao Hospital since 2006. Previous reports have found that WCR demonstrates good results for the treatment of patients with psoriasis. Among 136 Thai psoriasis patients who received WCR, 92.80% responded well. Although WCR is effective, there is still a lack of scientific data, especially relating to the bioactive compound in WCR. Therefore, this study aims to evaluate the phytochemicals in WCR via bioassay-guided isolation. **Methods**: In this study, the WCR was extracted via decoction with water, in a process based on traditional Thai medicine. The water extract was concentrated and dried using a spray dryer. The crude water extract was isolated using the partition technique with organic solvents, namely petroleum ether and ethyl acetate. These fractions were then separated and tested for anti-inflammatory activity using the bioassay-guided fractionation method. **Results**: Two particular types of pro-inflammatory cytokines are involved in inflammation and are among the factors that cause psoriasis—TNF-*α* and IL-6. Thus, we evaluated the isolated samples in terms of anti-inflammatory activity. The isolation resulted in two pure compounds—*p*-coumaryl aldehyde and *trans*-*p*-coumaryl alcohol. In the efficacy test of the isolated compounds, compared to the standard indomethacin at the same concentration of 12.5 ug/mL, *trans*-*p*-coumaryl alcohol was found to have the best efficacy, inhibiting TNF-*α* by 29.28% and IL-6 by 36.75%, with the standard compound showing inhibitions rates of 15.80% for TNF-*α* and 27.44% for IL-6. **Conclusions**: This study is the first report to identify the bioactive compound of WCR as *trans*-*p*-coumaryl alcohol or 4-hydroxycinnamyl alcohol.

## 1. Introduction

Psoriasis is a persistent inflammatory autoimmune skin disorder that affects approximately 2–5% of the global population. It is increasingly recognized for its systemic impact and comorbidities [1,2]. The condition is characterized by erythematous plaques with silver-white scales and is associated with reduced quality of life and increased risk of cardiovascular diseases, malignancies, and depression [2,3] The large-scale global study assessed psoriasis prevalence, healthcare access, and quality of life impacts using consistent physician-confirmed diagnoses across 20 countries covering over 50% of the world’s population. It found that Global prevalence of psoriasis is 4.4%, with East Asia having the highest (5.7%) and Africa the lowest (1.7%). Contrary to prior reports, Asia shows higher prevalence, and globally, men have slightly higher prevalence than women. Many patients, especially in North America, do not consult healthcare professionals, with dermatologist visits being lowest in Australia and North America, likely due to higher healthcare costs. Younger patients report more embarrassment and lifestyle limitations due to psoriasis, including avoiding social and professional activities. [4]. Psoriasis is becoming an increasingly significant health issue in Thailand, with over 20,000 outpatient visits recorded in 2024, making it the country’s third most prevalent dermatological condition. An estimated 1.7 million individuals are affected, and about 30% of them experience severe symptoms that often require advanced therapeutic approaches beyond conventional treatments. The average treatment cost per visit is approximately 3100 THB [5]. Standard treatments include topical corticosteroids, phototherapy, and systemic immunomodulators; however, many of these therapies are limited by significant side effects and high costs [6]. As a result, there is a growing interest in traditional medicine-based therapies, such as the Wannachawee recipe (WCR), which has demonstrated high clinical response rates and is used in various Thai traditional medicine [7,8] Wannachawee (WCR), has been used to treat psoriasis since 2006 in the Thai Traditional Medicine Clinic at Prapokklao Hospital. In a study of 136 Thai patients, 92.8% showed improvement following WCR treatment. WCR is composed of eight Thai herbs including Kha (*Alpinia galanga* (L.) Willd.), Khao Yen Tai (*Smilax glabra* Wall.ex Roxb.), Khao Yen Nuea (*Smilax corbularia* Kunth.), Khao Yen Jeen (*Smilax* sp.), Hua Ta Pead (*Stemona involuta* Inthachub.), Non Tai Yak (*Stemona collinsae* Craib.), Thong Pan Chang (*Rhinacanthus nasutus* (L.) Kurz.), and Ngueak plaamo (*Acanthus ilicifolius* L.) [8]. The herbs in WCR possess pharmacological activities that align with psoriasis symptoms, including anti-inflammatory, anti-proliferative, anti-angiogenic, antioxidant, and anti-tumor activities [9,10,11]. The pathophysiology of psoriasis consists of two phases. In the early stages of psoriasis pathogenesis, an initial trigger such as skin injury, infection, stress, etc., activates innate immune cells, including plasmacytoid dendritic cells (pDCs), natural killer cells, and keratinocytes. These activated cells release cytokines like interferon-alpha (IFN-α), tumor necrosis factor-alpha (TNF-α), interleukin-1 beta (IL-1β), and interleukin-6 (IL-6) which stimulate myeloid dendritic cells (mDCs). The mDCs then migrate to regional lymph nodes, where they present antigens and secrete IL-12 and IL-23, promoting the differentiation of naive T cells into T helper 17 (Th17) and T helper 1 (Th1) cells. The effector T cells eventually migrate to the skin tissue, releasing cytokines such as IL-17A, IL-17F, IL-22, IFN-γ, and TNF-α that activate keratinocytes. The activated keratinocytes proliferate and produce antimicrobial peptides, pro-inflammatory cytokines (IL-1β, TNF-α, IL-6), and chemokines, perpetuating a feedback loop that sustains chronic inflammation and leads to plaque formation. This cycle involves additional interactions with fibroblasts and endothelial cells, contributing to the ongoing immunopathological progression of psoriasis [3,12,13]. Plaque psoriasis accounts for roughly 80% to 90% of all psoriasis cases [14]. It is marked by an inflammatory pathway involving TNF-α, IL-23, and Th17 cells. Cytokines like IL-6, IL-1β, and IL-23 are key in activating Th17 cells, which contribute to the development of chronic inflammation and autoimmunity [3]. Another study on IL-6 states that serum IL-6 levels are considered a marker of inflammatory activity in psoriasis and a useful indicator of treatment response [15]. IL-6 in particular has emerged as both a biomarker for disease activity and a potential therapeutic target [16]. Clinical studies have reported that patients with psoriasis exhibited significantly elevated serum levels of TNF-α, averaging 362.67 ± 456.9 pg/mL, compared to 14.64 ± 5.78 pg/mL in healthy controls [17]. In patients with psoriasis, serum IL-6 levels ranged from 7 to 183 pg/mL, with a mean of 61.26 ± 57.40 pg/mL. In contrast, healthy controls exhibited a mean IL-6 concentration of 2.38 pg/mL [17]. This substantial difference was found to be highly statistically significant (*p* < 0.001), underscoring TNF-α and IL-6′s role as key pro-inflammatory cytokines in the pathogenesis of psoriasis. Based on these data and available resources, we chose to investigate the anti-inflammatory activity against pro-inflammatory cytokines TNF-α and IL-6. Despite the widespread use of Wannachawee recipe (WCR) in psoriasis treatment, scientific evidence supporting its efficacy, especially in terms of identifying active anti-inflammatory compounds, remains limited. There is also a lack of standardized chemical quality control for this traditional formulation, which poses challenges for broader acceptance and consistent clinical application.

## 2. Materials and Methods

### 2.1. Chemicals

Acetonitrile of HPLC grade, chloroform, deionized water (HDPE), ethyl acetate, hexane, methanol, and sulfuric acid of AR grade were purchased from RCI Labscan (V.S. CHEM HOUSE, Bangkok, Thailand). Celite was obtained from the Fluka Company (SIGMA-ALDRICH, Singapore). TLC Silica gel 60 F254, the aluminum support, and silica gel were acquired from Merck (Merck KGaA, Darmstadt, Germany).

### 2.2. Plant Materials

All crude drugs included in WCR were sourced from Prapokklao Hospital in Chanthaburi Province, Thailand. The plant materials were identified by comparison with the authentic samples found in a botanical herbarium at the Faculty of Pharmacy, Chiang Mai University or referenced in official manuscripts/monographs/previous report [12]. Details are presented in Table 1.

The samples were then cleaned, reduced in size by coarsely grinding them, and dried at 50 °C in a hot air oven (Scientific Promotion, Bangkok, Thailand) for 2 h.

### 2.3. Sample Preparation

The extraction process utilized traditional methods, specifically boiling herbs in water [14]. The coarse powder of WCR was extracted through decoction for 30 min, using water as the solvent (640 g WCR extract per 5L water; temperature 80–100 °C). The resulting water extract was filtered via steel grating and was concentrated to achieve a Brix value of 3–5, followed by drying using a spray dryer (BUCHI, Bangkok, Thailand).

### 2.4. Bioassay-Guided Isolation with Anti-Inflammation Analysis

The extract of WCR was isolated by column chromatography (BECTHAI Bangkok Equipment & Chemical, Bangkok, Thailand). The bioactive compound(s) were investigated following bioassay-guided fractionation.

#### 2.4.1. Separation Technique

Wannachawee extract was separated using column chromatography (CC) with silica gel as the stationary phase and a gradient mobile phase. The resulting fractions were examined by thin-layer chromatography (TLC) to identify and group similar chemical patterns. Subsequently, the anti-inflammatory activity of the fractions was tested. The active fractions were re-chromatographed by column chromatography and prep-TLC to obtain pure compound(s). A marker was selected from the bioactive compounds based on its high potency and significant concentration in the extract.

#### 2.4.2. Anti-Inflammatory Activity Test [18,19]

1. Cell used and Cell Culture

The RAW 264.7 macrophage cell line was chosen as the primary in vitro model because it is widely used to study the inflammatory cascade, particularly in response to LPS stimulation. It produces pro-inflammatory cytokines such as TNF-α and IL-6, which are central to early-stage inflammation and are well-established biomarkers in initial anti-inflammatory screenings, including psoriasis-related studies [20]. The cells were maintained in Dulbecco’s Modified Eagle’s Medium (DMEM; GIBCO™, Thermo Fisher Scientific, MA, USA) supplemented with 10% heat-inactivated fetal bovine serum (GIBCO™, Thermo Fisher Scientific, Waltham, MA, USA), 100 units/mL penicillin, and 100 μg/mL streptomycin (GIBCO™, Thermo Fisher Scientific, MA, USA). Cultures were kept in a CO_2_ incubator at 37 °C with 5% CO_2_.

2. Cytotoxicity Assay with Sulforhodamine B (SRB Assay)

RAW 264.7 cells (5 × 10^4^ cells/well) were seeded in a 96-well plate and incubated overnight at 37 °C in a 5% CO_2_ incubator. WCR extracts, prepared in various concentrations and dissolved in DMSO, were added to the cells which were then cultured for an additional 24 h. After this incubation, 10% trichloroacetic acid was added to fix the cells for 1 h at 4 °C. The cells were subsequently stained with 0.057% SRB for 30 min. The plate was washed with 1% acetic acid to remove excess dye and allowed to air dry at room temperature. To dissolve the protein-bound dye, Tris-base solution (10 mM) was added, and absorbance was measured at 510 nm using an ELISA plate reader. At least three concentrations were tested, and the final DMSO concentration was maintained below 0.1%. All experiments were conducted in triplicate and repeated in three independent experiments. Data were analyzed using GraphPad Prism version 9.0 to calculate means and standard errors.

3. Studying the effect of the extracts on the inhibition of TNF-*α* and IL-6

RAW 264.7 cells were cultured in a 96-well plate (5 × 10^4^ per well). Cells were incubated at 37 °C for 24 h. Then, three concentrations of herbal extracts were added to the well and incubated for 1 h. Lipopolysaccharide (Sigma-Aldrich, St. Louis, MI, USA) was added at a final concentration of 1 µg/mL to stimulate the cells. The cells were incubated for another 24 h. At the end of the incubation, culture supernatants were aspirated to quantify TNF-*α* and IL-6 by using ELISA (BioLegend, San Diego, CA, USA), as specified in the test kit. In this trial, indomethacin was used as the reference drug.

4. Statistical Analysis

All results are presented as the mean ± standard error of the mean (SEM) from three independent experiments. Statistical comparisons between groups were analyzed using one-way analysis of variance (ANOVA), followed by Tukey’s post hoc test for multiple comparisons. A *p*-value of less than 0.05 was considered statistically significant. The kurtosis statistic was used to assess the normality of the data distribution, where values within ±3 were considered acceptable for applying parametric tests. All analyses were performed using GraphPad Prism version 9.0.

### 2.5. Identification of Chemical Compounds in WCR Extract by HPLC

The Shimadzu Prominence High-Performance Liquid Chromatography system (SpectraLab Scientific Inc., Markham, ON, Canada), comprising a DGU-20A3R degasser, 2x LC-20AD liquid chromatographs, an SIL-20A auto-sampler, a CTD-20A column oven, and a SPD-20A UV-Visible detector, was used. The column used was a Mightysil, RP-C18 GP (250×4.6 mm,5 µm) equipped with a guard column. The mobile phase was a gradient prepared from 10% (*v/v*) acetonitrile (component A) and formic acid in HPLC-grade water (component B), as detailed in Table 2, with a flow rate of 1.0 mL/min. The absorption wavelength was selected at 254 nm. The column was operated at 25 °C. The sample injection volume was 20 µL.

### 2.6. Structure Elucidation by Spectroscopy

The structure of the isolated compound was elucidated using a Bruker NEOTM 500 MHz NMR spectrometer by measuring ^1^H NMR, ^13^C NMR, COSY, HMQC, and HMBC. Structure analysis was performed with MesReNova.

## 3. Results

### 3.1. Preparation of the Extract

The extraction process followed the traditional method, which is known as decoction. The total volume obtained was subsequently processed using spray drying. The results are presented in Table 3.

### 3.2. Bioassay-Guided Fractionations

#### 3.2.1. Separation Results

**Figure 1:**
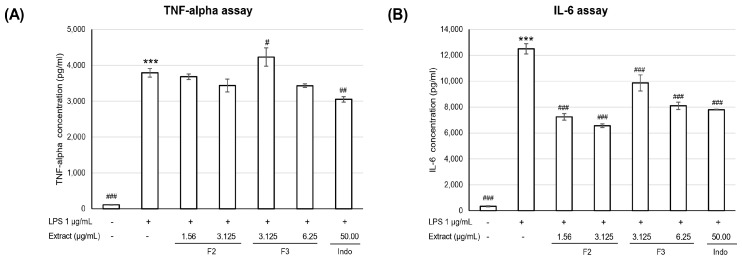
Effect of F2 and F3 of EtOAc fraction of WCR extract on inhibition of TNF-*α* (**A**) and IL-6 (**B**) at 24 h. Data are presented as mean ± SEM from three independent experiments. # Statistically significantly different from unstimulated cells, # *p* < 0.05, ## *p* < 0.01, ### *p* < 0.001. * Statistically significantly different from LPS-stimulated cells, *** *p* < 0.001.

The crude extract of WCR was isolated using a partition technique, resulting in two fractions: ethyl acetate (EtOAc) and an aqueous fraction. The EtOAc fraction demonstrated the most potent activity. At a concentration of 3.125 µg/mL, the EtOAc fraction reduced the levels of TNF-α and IL-6 to 5555.34 ± 372.59 pg/mL pg/mL and 3765.62 ± 504.73 pg/mL, respectively. In comparison, the standard drug indomethacin, administered at a dose of 50 µg/mL, inhibited TNF-α to 4035.67 ± 191.57 pg/mL and IL-6 to 3543.95 ± 204.87 pg/mL. The EtOAc fraction was then subjected to column chromatography, yielding 15 subfractions. It was observed that F2 and F3 fractions, at the highest concentration, caused a slight decrease in TNF-α levels (Figure 1A). Meanwhile, F2 and F3 fractions at all concentrations resulted in a statistically significant decrease in IL-6 levels. Notably, cells incubated with the F2 fraction at all concentrations decreased the amount of IL-6 more effectively than those incubated with indomethacin (Figure 1B).

Then, F2 was re-chromatographed to afford 17 subfractions (F2A-F2P). At the highest concentrations of fractions F2A, F2E, and the EtOAc extract, the levels of TNF-α were measured at only 2215 ± 153 pg/mL, 2600 ± 363 pg/mL, and 2611 ± 446 pg/mL, respectively, after incubating the cells with the herbal extract. These results align with a corresponding decrease in IL-6 levels. It was observed that at the highest concentrations, F2A, F2E, and the EtOAc extract were the most effective in reducing IL-6 levels (Table 4).

**Figure 2:**
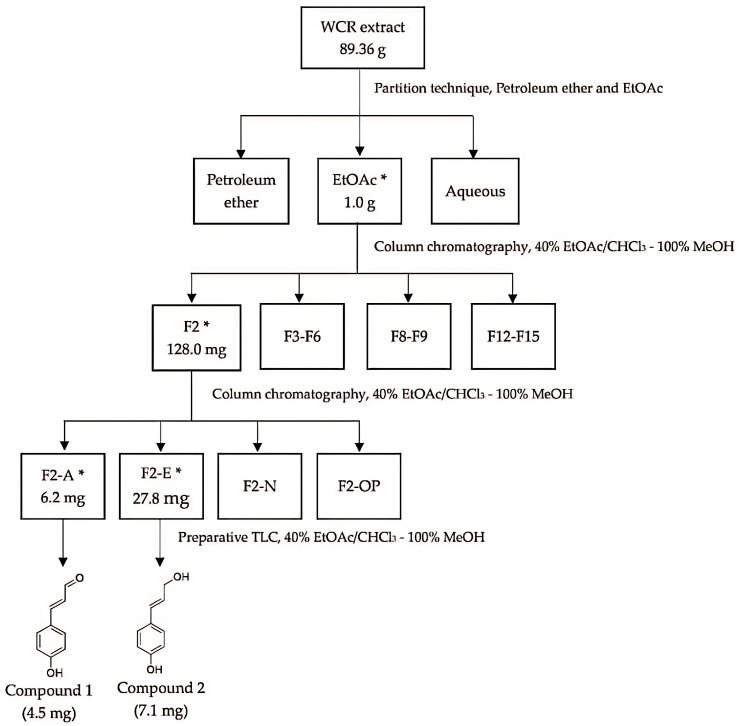
Bioassay-guided fractionation of crude Wannachawee (WCR) extract and its anti-inflammatory activity against TNF-α and IL-6 (* = active fraction).

Additionally, fractions F2A and F2E were separated using preparative thin-layer chromatography (Prep-TLC), resulting in two pure compounds, as shown in Figure 2, i.e., *p*-coumaryl aldehyde and *trans*-*p*-coumaryl alcohol.

#### 3.2.2. Anti-Inflammatory Activity

1. Cytotoxicity assay

**Figure 3:**
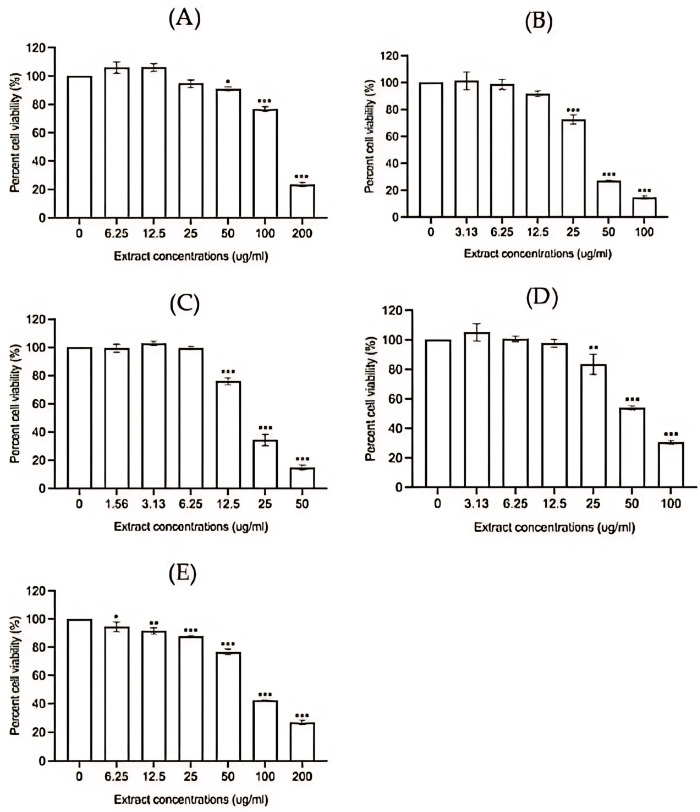
Effects of pure compound 1 (**A**), pure compound 2 (**B**), F2 (**C**), EtOAC fraction (**D**), and indomethacin (**E**) on growth of RAW 264.7 cells at 24 h. Data are presented as mean ± SEM from three independent experiments. Values are significantly different from control cells. * *p* < 0.05, ** *p* < 0.01, and *** *p* < 0.001.

The isolated compounds, the EtOAc fraction, and F2 were selected to assess their anti-inflammatory activity in RAW 264.7 cells. The cytotoxicity assay demonstrated significant cell death at 24 h after exposure to various concentrations of the test extracts. Noticeable cell death was observed at the following concentrations: compound 1 at 50–200 µg/mL (Figure 3A), compound 2 and the EtOAc extract at 25–100 µg/mL (Figure 3B,D), and F2 at 12.5–50 µg/mL (Figure 3C). For indomethacin, a standard anti-inflammatory drug, statistically significant cell death occurred at concentrations of 6.25 µg/mL or higher (Figure 3E). The cytotoxicity exhibited a clear concentration-dependent pattern.

2. Effect of extracts on inhibition of TNF-*α* and IL-6

**Figure 4:**
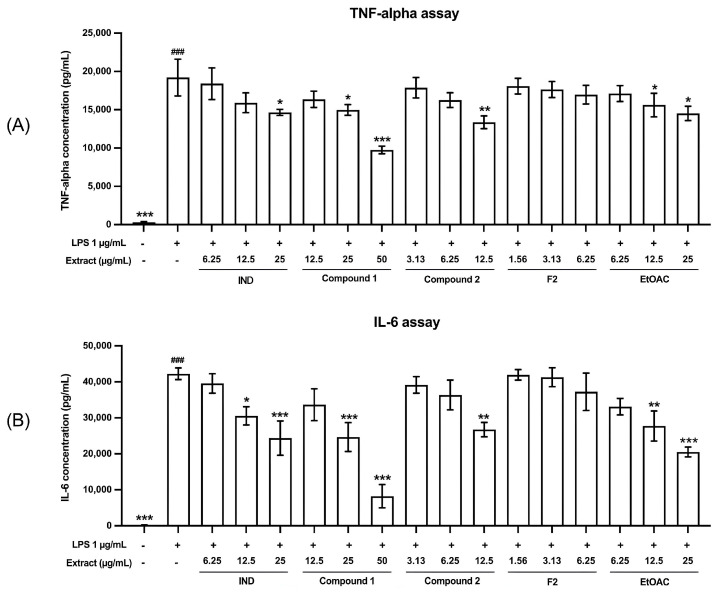
Effect of test compounds and fractions on the inhibition of TNF-*α* (**A**) and IL-6 (**B**) at 24 h. Data are presented as mean ± SEM from three independent experiments. # Statistically significantly different from unstimulated cells; ###: *p* < 0.001. * Statistically significantly different from LPS-stimulated cells; *: *p* < 0.05; **: *p* < 0.01; and ***: *p* < 0.001. InD: indomethacin.

The anti-inflammatory activity of the pure compounds and fractions was assessed in LPS-stimulated RAW 264.7 cells by measuring the level of TNF-*α* and IL-6 using the ELISA kit. The results indicate that after LPS stimulation, cells produced significantly higher levels of TNF-*α* and IL-6, with concentrations of 19,200 ± 2402 and 42,257 ± 1623 pg/mL, respectively, compared to unstimulated cells. When the extracts were added at various concentrations to assess their anti-inflammatory activity, both pure compound 1 and pure compound 2 significantly reduced TNF-*α* levels, as shown in Figure 4A. Furthermore, pure compound 1, pure compound 2, and the EtOAc extract also significantly decreased IL-6 levels, as shown in Figure 4B. The anti-inflammatory effects of these test compounds were comparable to those of indomethacin, which is the positive control used in this study.

**Figure 5:**
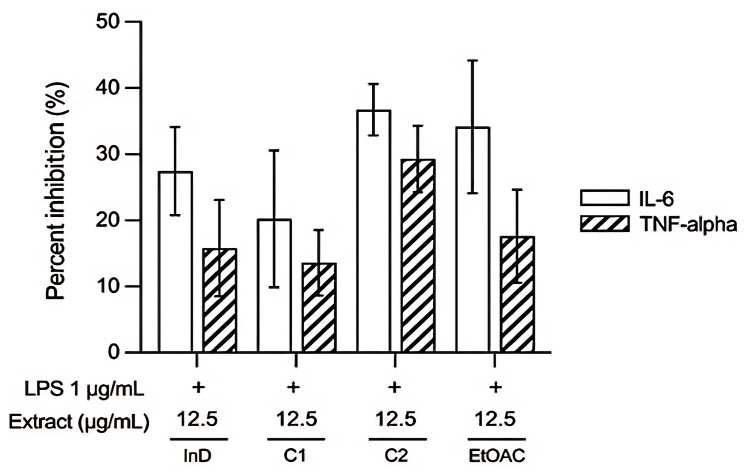
The effects of the test compounds and fractions at equal concentrations on anti-inflammatory activity at 24 h. The anti-inflammatory activity was directly proportional to the percentage of TNF-*α* and IL-6 inhibition. The data are presented as the mean ± SEM from 3 independent experiments. *InD*: indomethacin; *C1*: pure compound 1; *C2*: pure compound 2.

When comparing the anti-inflammatory activity of the test substances at the same concentration (12.5 µg/mL), it was found that pure compound 2 and the EtOAc extract exhibited better inhibition of inflammation than indomethacin, with consistent results for both TNF-*α* and IL-6 inhibition, as shown in Figure 5. The most effective extract in inhibiting inflammation was pure compound 2, which inhibited TNF-*α* by 29.28 ± 5.02% and IL-6 by 36.75 ± 3.88%. This was followed by EtOAc, which inhibited TNF-*α* by 17.59 ± 7.05% and IL-6 by 34.16 ± 10.04%, and then pure compound 1, which inhibited TNF-*α* by 13.59 ± 4.95% and IL-6 by 20.24 ± 10.34%.

### 3.3. Chemical Identification of WCR Recipe by HPLC

**Figure 6:**
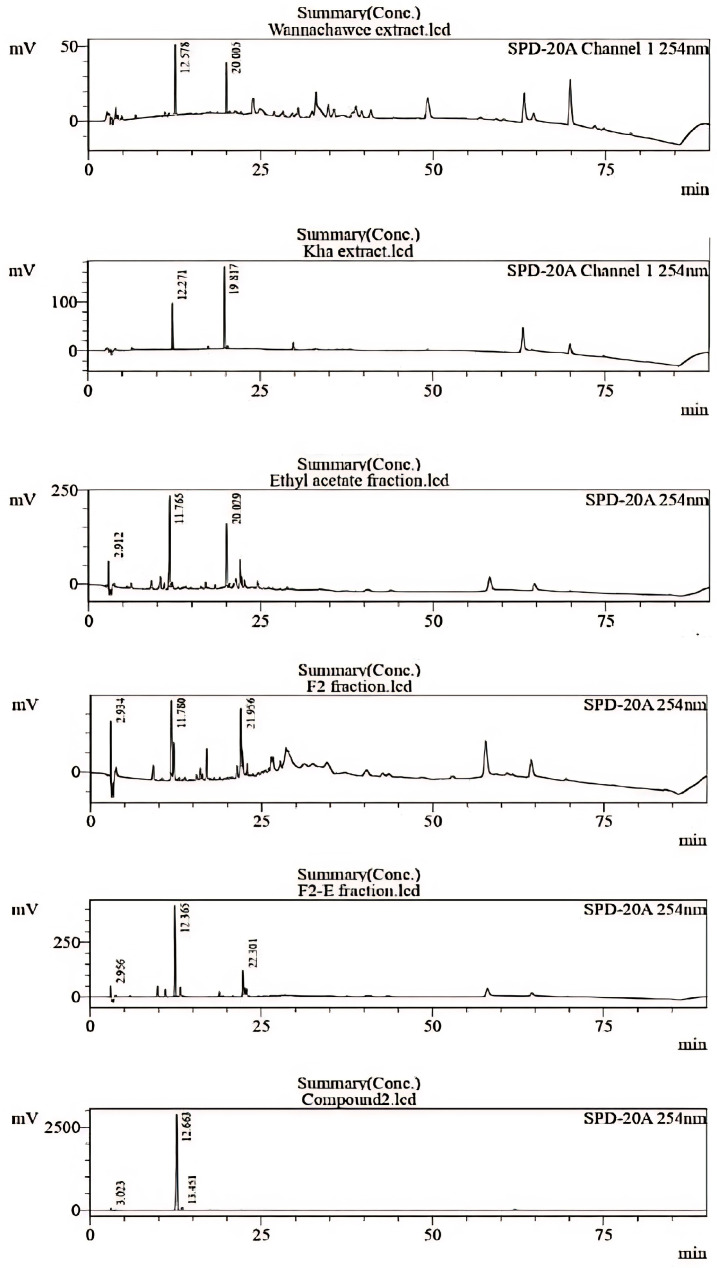
Chromatogram of Kha extract, EtOAc fraction, F2 fraction, F2-E fraction, and compound 2 at 254 nm in WCR extract.

HPLC is an effective method for ensuring the chemical quality of herbal extracts. Comparing the chemical constituents in the WCR extract with each herb and other bioactive fractions revealed interesting peaks at specific time points (Rt = 12, 20 min). The chromatograms of the Kha extract displayed the same Rt with the main compound in WCR recipe. Thus, this crude extract was separated. The chromatograms of the Kha (*A. galanga*) extract, EtOAc fraction, F2 fraction, F2-E fraction, and compound 2 are shown in Figure 6. The anti-inflammatory activity was primarily attributed to the ethyl acetate fraction. After purification, fraction F2 gave two pure compounds. One of these was identified as *trans*-*p*-coumaryl alcohol (Rt = 12 min), which demonstrated potent activity and was the predominant compound.

### 3.4. Results of Structure Elucidation

**Figure 7:**
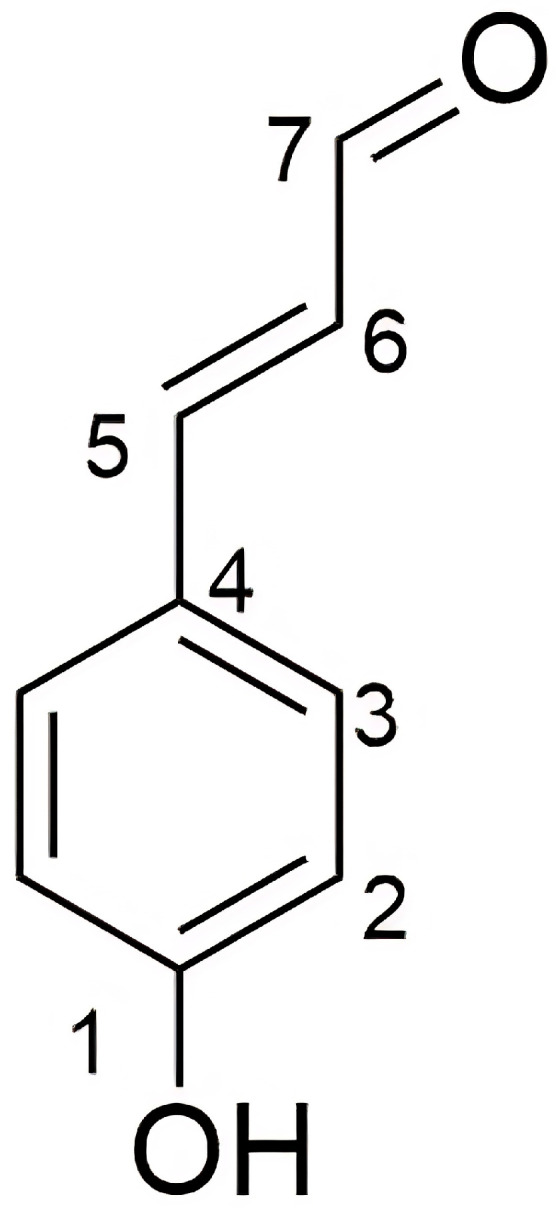
The position and structure of compound 1 (*p*-coumaryl aldehyde).

Regarding its physical characteristics, compound 1 (4.5 mg) is a brown powder. The structural elucidation of compound 1 was performed using a nuclear magnetic resonance spectrometer (NMR) for ^1^H NMR and ^13^C NMR data, as shown in Table 5 and Figure 7.

The ^1^H-NMR spectrum for compound 1 shows one signal of aldehyde proton [δ8.54 (*s*, 1H)], two signals of p-disubstituted aromatic protons [δ7.24 (*d*, 8.5 Hz, 2H)] and [δ*6*.73 (*d*, 8.5 Hz, 2H)], and two signals of trans-olefinic proton [δ 6.52 (*d*, 16.0 Hz, 1H)] and [δ 6.11 (*dt*, 15.5, 6.5 Hz, 1H)]. The ^13^C-NMR spectrum shows six signals of eight carbons including one signal of aldehyde carbon [δ200.08 (C-7)], two signals of quaternary carbon [δ180.3 (C-4) and 159.3 (C-1)], two signals of four aromatic methine carbons [δ128.8 (2C) and 116.4 (2C)], and two signals of olefinic methine carbon [*δ*133.9 (C-5) and 122.4 (C-6)]. According to the Scifinder database, compound 1 is a structurally reported substance called *p*-coumaryl aldehyde [22] or *p*-hydroxycinnamaldehyde [23].

**Table 5:** ^1^H and ^13^C-NMR data of compound 1 (Methanol-*d_4_*, ^1^H NMR 500 MHz, ^13^C NMR 125 MHz).

Position	Compound 1		*p*-Coumaryl Aldehyde
	*δ_H_* (*mult*., *J* in Hz)	δ*_c_*, *mult*	δ*_H_* (*mult*., *J* in Hz) of Ref. [23]
1	-	159.3, C	-
2	6.73 (*d*, 8.5, 2H)	116.4, CH	6.72 (*d*, 8.5, 2H)
3	7.27 (*d*, 8.5, 2H)	128.8, CH	7.24 (*d*, 8.5, 2H)
4	-	180.3, C	-
5	6.50 (*d*, 16.0, 1H)	133.9, CH	6.50 (*d*, 15.9, 1H)
6	6.11 (*dt*, 15.5, 6.5, 1H)	122.4, CH	6.16 (*dt*, 15.9, 6.0, 1H)
7	8.54 (*s*, 1H)	200.08, CHO	9.56 (*d*, 7.9, 1H)

*s* = singlet; *d* = doublet; *dd* = double of doublet; *dt* = double of triplet.

**Figure 8:**
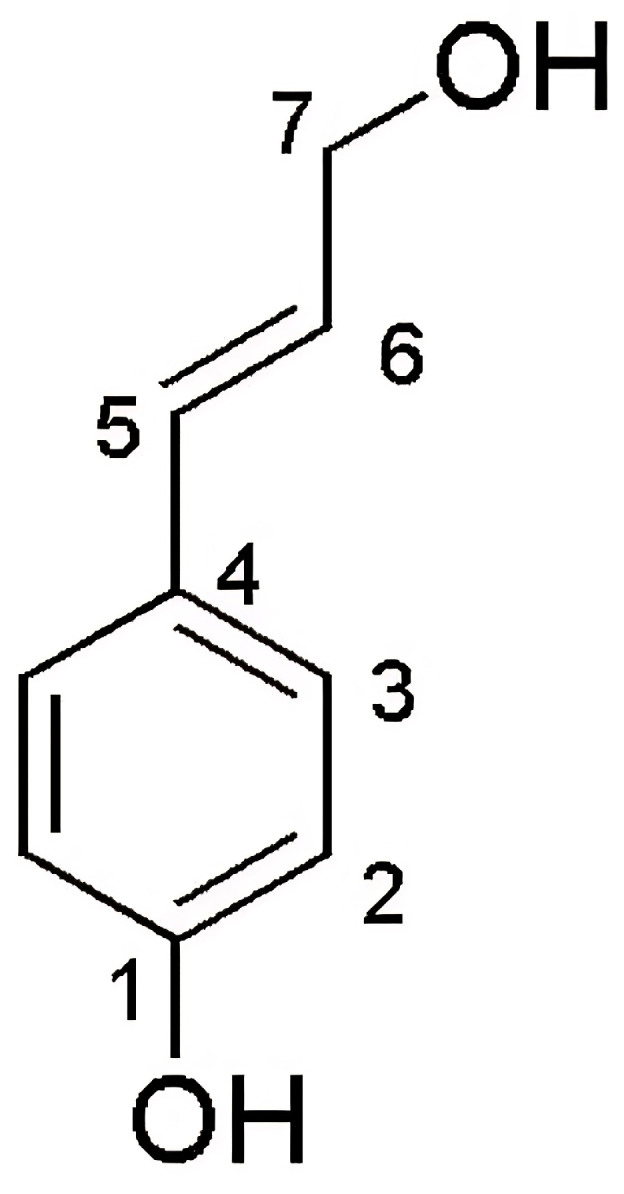
The position and structure of compound 2 (*trans*-*p*-coumaryl alcohol).

Regarding its physical characteristics, compound 2 (7.1 mg) is a brown powder. The structural elucidation of compound 2 was performed using a nuclear magnetic resonance spectrometer (NMR) for ^1^H NMR and ^13^C NMR data, as shown in Table 6 and Figure 8.

The ^1^H-NMR spectrum shows two signals of p-disubstituted aromatic protons [δ*7*.27 (*d*, 8.5 Hz, 2H)] and [δ6.72 (*d*, 8.5 Hz, 2H)], two signals of trans-olefinic proton [δ6.50 (*d*, 16.0 Hz, 1H)] and [δ6.16 (*dt*, 16.0, 6.0 Hz, 1H)], and one signal of methylene protons [δ4.18 (*dd*, 6.0, 1.5 Hz, 2H)]. The ^13^C-NMR spectrum data show that compound 2 has seven signals of nine carbons including two signals of quaternary carbon [δ158.2 (C-1) and 129.9 (C-4)], two signals of four aromatic methine carbons [δ128.6 (2C) and 116.3 (2C)], two signals of olefinic methine carbon [*δ*131.8 (C-5) and 126.6 (C-6)], and one signal of methylene carbon [δ63.9 (C-7)]. According to the Scifinder database, compound 2 is a structurally reported substance called trans *p*-coumarin alcohol [24] or 4-hydroxycinnamyl alcohol [25].

## 4. Discussion

Wannachawee recipe (WCR) is a traditional Thai medicine that has been used for generations to treat psoriasis. Since 2006, WCR has been utilized at the Thai Traditional Medicine Clinic of Prapokklao Hospital. This initiative is the result of collaboration between practitioners of modern medicine and those of traditional Thai medicine. In a study of 136 Thai psoriasis patients receiving WCR, 92.80% showed a good response [7,8]. Psoriasis is an immune-mediated chronic inflammatory skin disease [26]. There is evidence that inflammation drives immune and metabolic changes that exacerbate psoriasis [27]. One of the characteristics of chronic inflammation is the production of inflammatory cytokines [28]. TNF-α and IL-6 are the important cytokines involved in inflammation in psoriasis [3,14,15]. A previous report showed that TNF-α and IL-6 levels are higher in psoriasis patients compared to healthy individuals [17]. Therefore, the levels of these two cytokines are associated with psoriasis, making them reasonable markers for screening. Although WCR is continually used, there is still limited information available regarding chemical quality control. In our research, we aimed to study the anti-inflammatory properties of the bioactive compounds which can support biomarker selection in quality control processes and traditional Thai applications.

All crude drugs listed in WCR were obtained from Prapokklao Hospital in Chanthaburi Province, Thailand. The crude drugs used in this study are commonly utilized in hospitals. Identification was conducted by a Thai practitioner who specializes in Thai herbal medicine, as well as through comparison with authentic samples from a botanical herbarium or descriptions in manuscripts, monographs, or previous reports [29]. Traditional use of WCR involves extracting water through boiling. The decoction process is employed to align with traditional medicine, yielding 14.34% *w/w*. Bioassay-guided isolation was employed to identify the bioactive compound(s). *Trans*-*p*-coumaryl alcohol and *p*-coumaryl aldehyde were identified as the bioactive compounds.

Previous reports have indicated that these two compounds were isolated from the rhizomes and fruits of *A. galanga* in the ethyl acetate (EtOAc) fraction using reversed-phase ODS column chromatography [30,31,32,33]. Additionally, it was noted that *p*-coumaryl aldehyde was isolated from the 95% ethanol and hot water fractions of *A. galanga* using column chromatography [34]. In a study on the inhibition of melanogenesis in B16 melanoma 4A5 cells induced by theophylline, *p*-coumaryl aldehyde had an IC_50_ value of 18.3 µM, while *trans*-*p*-coumaryl alcohol had an IC_50_ value > 100 µM [34]. Both substances are effective neuroprotective agents due to their good permeability [34]. *p*-Coumaryl aldehyde has also been reported in *Sarcophyta sanguinea* [22]. It has also been reported to exhibit antimicrobial activity [22], while *trans*-*p*-coumaryl alcohol is a component of *A. officinarum* [33] and *Malus domestica* [35]. Previous research reports have shown that this compound demonstrates antioxidant activity by inhibiting the autoxidation of methyl linoleate in the bulk phase [35]. The two compounds identified in the extract of *A. galanga* align with the HPLC chromatograms. The peak for *trans*-*p*-coumaryl alcohol, which is a major component, appears at 12 min, corresponding to the *A. galanga* extract (see Figure 6). Additionally, *p*-coumaryl aldehyde is present as a minor compound, observed at 25 min. It has been established that *trans*-*p*-coumaryl alcohol is one of the major ingredients in the *A. galanga* water extract. In previous reports, three compounds from the genus *Alpinia*, namely (1E,6E)-1,7-Bis(4-hydroxy-3-methoxyphenyl)hepta-1,6 diene-3,5-dione, (−)-alphainanins A, and (−)-alphainanins C, exhibited anti-inflammatory mechanisms by inhibiting the production of TNF-α, IL-6, NO, IL-1β, and NO via the NF-kB signaling pathway in RAW264.7 cells upon inflammatory stimulation with LPS [33]. This indicates that the extract from *A. galanga* may also have anti-inflammation activity. Due to its potency and high concentration in the WCR extract, *trans*-*p*-coumaryl alcohol was chosen as a biomarker. The HPLC technique is an effective method for assessing the quality of extracts and products, including the monitoring of their stability through their biomarker.

In this study, the focus was on early-stage anti-inflammatory screening, specifically targeting immune-mediated pathways relevant to psoriasis pathogenesis. The RAW 264.7 macrophage cell line was selected as an appropriate in vitro model for this phase due to its well-established role in inflammation research. Upon stimulation with lipopolysaccharide (LPS), RAW 264.7 cells reliably produce key pro-inflammatory cytokines, making this model a widely accepted and reproducible platform for initial screening of anti-inflammatory activity. This study shows that *trans*-*p*-coumaryl alcohol and *p*-coumaryl aldehyde exerted anti-inflammatory effects. *Trans*-*p*-coumaryl alcohol exhibited anti-inflammatory properties by inhibiting TNF-α and IL-6 in RAW 264.7 macrophage cells. *Trans*-*p*-coumaryl alcohol demonstrated greater activity than the reference drug, indomethacin, while *p*-coumaryl aldehyde exhibited lower activity at the same concentration. Indomethacin was selected in this study as a standard reference due to its well-characterized anti-inflammatory properties. It is widely employed in in vitro inflammation models, particularly with LPS-stimulated RAW 264.7 macrophage cells, to benchmark the inhibition of pro-inflammatory cytokines such as TNF-α and IL-6. These cytokines play central roles in the pathogenesis of psoriasis [10]. Therefore, indomethacin was used as a positive control to compare the anti-inflammatory efficacy of WCR extracts and isolated compounds under standardized conditions.

To evaluate the therapeutic potential of the isolated compounds, we compared their cytotoxicity and anti-inflammatory efficacy. *Trans-p*-coumaryl alcohol significantly inhibited TNF-α and IL-6 production at 12.5 µg/mL, while cytotoxicity was observed at concentrations ≥25 µg/mL, with an estimated IC_50_ of approximately 50 µg/mL. This yields a preliminary therapeutic index (TI) of about 4, suggesting a favorable safety margin in this in vitro setting. A similar pattern was observed for the EtOAc fraction, whereas p-coumaryl aldehyde exhibited a narrower margin.

*Trans*-*p*-coumaryl alcohol and *p*-coumaryl aldehyde are phenylpropanoid compounds. Phenylpropanoids can inhibit inflammation through multiple mechanisms, such as suppression of TNF-*α*, IL-1β, and IL-6 production, suppression of NF-kB, reduced release of ROS, and prevention of histamine-induced bronchoconstriction [36]. In addition, *p*-coumaryl aldehyde and *trans*-*p*-coumaryl alcohol are also substances in the phenolic compound group which have a mechanism of action that inhibits mediators in the inflammation process, such as IL-1β, IL-6, IL-8, TNF-*α*, ROS, nitric oxide (NO), and prostaglandins [37]. Cytokines such as IL-17, IL-23, and IL-36 are well-established mediators in the pathogenesis of psoriasis and play critical roles in sustaining the chronic inflammatory environment characteristic of the disease [38,39]. These cytokines have also emerged as key therapeutic targets in current clinical strategies for psoriasis management [40,41]. The progression of psoriasis involves two major phases: an initial immune-mediated inflammatory phase followed by a keratinocyte hyperproliferation phase.

In the present study, we focused on the early inflammatory phase by assessing the inhibitory effects of the WCR extract on key pro-inflammatory cytokines, specifically TNF-α and IL-6. This approach provides initial mechanistic insight into the anti-inflammatory potential of the extract. While the LPS-stimulated RAW 264.7 macrophage model is widely utilized for preliminary anti-inflammatory screening, it primarily reflects an acute inflammatory response. Given that psoriasis is a chronic immune-mediated condition driven by the IL-23/Th17 axis, the use of LPS may not fully recapitulate its immunopathogenesis [42]. Therefore, future studies should include more disease-relevant models, such as IL-23-stimulated RAW 264.7 cells, to better represent the chronic inflammatory microenvironment and more accurately assess the therapeutic potential of the WCR extract in the context of psoriasis. For a more comprehensive understanding of WCR’s therapeutic potential, future studies should include evaluations of its anti-proliferative effects using relevant keratinocyte models such as HaCaT cells. Additionally, the investigation of a broader cytokine profile—including IL-17, IL-23, and IL-36—will be necessary to fully elucidate the underlying mechanisms and clinical relevance of WCR in psoriasis treatment.

## 5. Conclusions

This study successfully identified the bioactive compounds in Wannachawee recipe (WCR) using a bioassay-guided fractionation approach. Two phenylpropanoid compounds—*trans*-*p*-coumaryl alcohol and *p*-coumaryl aldehyde—were isolated from the ethyl acetate fraction of the WCR extract. Among them, *trans*-*p*-coumaryl alcohol demonstrated the most potent anti-inflammatory activity, significantly inhibiting the production of TNF-α (29.28%) and IL-6 (36.75%) in LPS-stimulated RAW 264.7 cells, surpassing the efficacy of the reference drug, indomethacin, at the same concentration.

These findings indicate that *trans-p*-coumaryl alcohol is a promising biomarker compound that can be used in relation to the quality control of WCR formulations. Moreover, this study provides scientific evidence supporting the traditional use of WCR for psoriasis treatment and lays the foundation for its standardization and further pharmacological validation.

## Figures and Tables

**Table 1:** Medicinal plants contained in Wannachawee recipe.

Thai Name	Scientific Name	Family	Part Used	Voucher No.
Kha	*Alpinia galanga* (L.) Willd.	Zingiberaceae	Rhizome	0023406
Khao YenTai	*Smilax glabra* Wall.ex Roxb.	Smilacaceae	Rhizome	0023412
Khao Yen Nuea	*Smilax corbularia* Kunth.	Smilacaceae	Rhizome	0023411
Khao Yen Jeen	*Smilax* sp.	Smilacaceae	Rhizome	0023413
Hua Ta Pead	*Stemona involuta* Inthachub.	Stemonaceae	Root	0023409
Non Tai Yak	*Stemona collinsae* Craib.	Stemonaceae	Root	0023408
Thong Pan Chang	*Rhinacanthus nasutus* (L.) Kurz.	Acanthaceae	Aerial part	0023410
Ngueak plaamo	*Acanthus ilicifolius* L.	Acanthaceae	Aerial part	0023407

**Table 2:** Gradient program for HPLC method (modified from [21]).

Time (min)	A (per cent *v/v*)	B (per cent *v/v*)
0.01	10	90
20.00	45	55
40.00	50	50
45.00	50	50
80.00	80	20
85.00	10	90
90.00	10	90

**Table 3:** The results of preparing the water extract of WCR.

Volume of conc. Water Extract (mL)	% Brix	pH	Dried Water Extract (g)	% Yield (*w*/*w*)
2900 *	3 *	5.17 *	91.81 *	14.34 *

* = single batch.

**Table 4:** Effect of F2 subfractions on levels of TNF-α and IL-6.

Extract Concentration (µg/mL)		TNF-α (pg/mL)	IL-6 (pg/mL)
Unstimulated cell		162 ± 25	285 ± 27
LPS-induced cell		3661 ± 170 ^###^	10,912 ± 856 ^###^
Ethyl acetate	3.13	2796 ± 281 *	6730 ± 956 ***
	6.25	2611 ± 446 **	5349 ± 1329 ***
F2-A	1.56	2443 ± 167 **	5055 ± 157 ***
	3.13	2215 ± 153 ***	3929 ± 128 ***
F2-E	1.56	2692 ± 200 **	5993 ± 557 ***
	3.13	2600 ± 363 **	5155 ± 524 ***
F2-N	12.5	2607 ± 335 **	5292 ± 1483 ***
	25	2625 ± 48 **	6041 ± 318 ***
F2-OP	12.5	2673 ± 146 **	6811 ± 123 ***
	25	2649 ± 281 **	6365 ± 454 ***
Indomethacin	25	1851 ± 170 ***	3910 ± 202 ***
	50	1705 ± 160 ***	3094 ± 195 ***

Data are presented as mean ± SEM from three independent experiments. # Statistically significantly different from unstimulated cells, ### *p* < 0.001. * Statistically significantly different from LPS-stimulated cells, * *p* < 0.05, ** *p* < 0.01, *** *p* < 0.001.

**Table 6:** ^1^H and ^13^C-NMR data of compound 2 (Methanol-*d_4_*, ^1^H NMR 500 MHz, ^13^C NMR 125 MHz).

Position	Compound 2		*trans*-*p*-Coumaryl Alcohol	
	δ*_H_ (mult*., *J* in Hz)	δ*_c_*, *mult*	δ*_H_* (*mult*., *J* in Hz) of Ref. [24]	δ*_c_*, (*mult*) of Ref. [23]
1	-	158.2, C	-	157.73, C
2	6.72 (*d*, 8.5, 2H)	116.3, CH	6.74 (*d*, 8.6, 2H)	116.29, CH
3	7.27 (*d*, 8.5, 2H)	128.6, CH	7.25 (*d*, 8.5, 2H)	128.31, CH
4	-	129.9, C		128.82, C
5	6.50 (*d*, 16.0, 1H)	131.8, CH	6.52 (*d*, 15.8, 1H)	129.59, CH
6	6.16 (*dt*, 16.0, 6.0, 1H)	126.6, CH	6.18 (*dt*, 15.8, 6.0, 1H)	128.08, CH
7	4.18 (*dd*, 6.0, 1.5, 2H)	63.9, CH_2_	4.20 (*dd*, 6.0, 1.4, 2H)	62.66, CH_2_

*s* = singlet; *d* = doublet; *dd* = double of doublet; *dt* = double of triplet.

## Data Availability

The original contributions presented in the study are included in the article, further inquiries can be directed to the corresponding author.
